# Supplementary material for: Mutations in bacterial genes induce unanticipated changes in the relationship between bacterial pathogens in experimental otitis media
Source: R Soc Open Sci. 2018 Nov 14;5(11):180810. doi: 10.1098/rsos.180810 (PMC6281918; doi:10.1098/rsos.180810)
Supplement: Supplementary Material 1 [file rsos180810supp1.pdf]

Supplementary Material for "*Mutations in bacterial genes induce unanticipated changes in the relationship between bacterial pathogens in experimental otitis media*"

1. Basic statistics of *in vivo* bacterial abundance data

2. Details of the MaxEnt calculation for estimation of $P(N_1,N_2)$ using the data for the wild type strains

3. Derivation of Stability Analysis

4. Two parameter marginal distributions of $Q(\alpha_{11}, \alpha_{12}, \alpha_{21}, \alpha_{22})$

5. Comparison between the Condorcet winner model and measurement at day 14 post inoculation with mutant strains

6. Doubling the range of the α-domain does not change the results

7. *In silico* Reference Data

8. Summary of all *in silico* mutant strain results

1. Basic statistics of *in vivo* bacterial abundance data

Table S1 shows some basic statistical analysis of all the *in vivo* data collected. The statistics include the mean, variance and covariance of the bacterial populations. *In vivo* data was collected at two time points: 7 and 14 days post inoculation. In addition to the wild type strains, various mutant strains were studied.

Table S1. Means, variances and covariances for the populations of NTHi and Mcat strains measured in chinchilla middle ears.

Co-infecting strains	Mean populations		Variances		Covariance	
	day 7	day 14	day 7	day 14	day 7	day 14
NTHi (WT) +	35.696	47.3587	1425.3	18210.5	0.0969	-1.2283
Mcat (WT)	0.1995	0.21223	0.0485	0.096923		
NTHi (WT) +	17.227	0.12875	1021.46	0.077510	0.000444	0.113955
Mcat (*hag*)	0.11512	0.02247	0.03328	0.000444		
NTHi (WT) +	88.5125	6.67173	13877.2	117.289	0.0046	23.0636
Mcat (*mcaB*)	0.41073	0.04287	0.90262	0.004638		
NTHi (WT) +	11.7292	4.67776	330.785	35.3254	1.7555	-0.06552
Mcat (*aaa*)	0.2902	0.49658	0.13586	1.75548		
NTHi (*luxS*) +	5.262	2.2379	150.58	62.0973	0.0029	-0.48528
Mcat (WT)	0.20410	0.03844	0.1717	0.002929		
NTHi (WT) +	7.369	0.17218	104.259	0.019129	0.0035	-1.03834
Mcat (*mclR*)	0.2742	0.03255	0.15760	0.003506		
NTHi (WT) +	2.10113	16.6376	24.7049	1115.9	0.000384	-0.07083
Mcat (*dtgt*)	0.04862	0.0168	0.00112	0.000384		
NTHi (*luxS*) +	37.9777	3.93157	3492.77	131.423	0.0012	-1.89478
Mcat (*mcaB*)	0.09526	0.03864	0.00826	0.001218		
NTHi (*luxS*) +	0.1805	0.1541	0.0433	0.0457	0.000598	-0.2104
Mcat (*hag*)	1.2638	0.0198	6.0147	0.000598		
NTHi (*luxS*) +	1.4704	0.1593	15.4776	0.1297	0.000524	-0.0502
Mcat (*dtgt*)	0.1	0.015	0.0083	0.000524		
NTHi (*luxS*) +	1.8746	0.8301	8.833	3.0997	0.0256	-0.4334
Mcat (*aaa*)	0.2436	0.1301	0.0817	0.0256		
NTHi (*luxS*) +	0.7194	2.3987	0.8152	22.2866	0.000368	-0.0613
Mcat (*mclR*)	0.1676	0.0166	0.0143	0.000368		

2. Details of the MaxEnt calculation for estimation of $P(N_1,N_2)$ using the data for the wild type strains

We used a MaxEnt approach to model the NTHi(WT) + Mcat(WT) co-infection data. Specifically, the $P(N_1,N_2)$ model was in the form

$$P(N_1, N_2) = \frac{1}{Z}\exp\left(-aN_1 - bN_2 - cN_1^{\ 2} - dN_2^{\ 2} - eN_1N_2\right)$$

Z is the normalization parameter. The remaining parameters were calculated such that the means of N_1 (*a*) and N_2 (*b*), variances of N_1 (*c*) and N_2 (*d*), and the covariance (*e*) matched the measured data. These five parameters in the exponent were numerically solved for using the 'fsolve' function in Matlab. We tried 100,000 random initial guesses for the parameters and chose the set which minimized the error in each of the five statistical measures mentioned above.

For day 7 NTHi(WT) + Mcat(WT), the parameters are

Z	28.7656
A	1.73908×10^{-8} CFU^{-1}
B	2.13905×10^{-6} CFU^{-1}
C	3.06493×10^{-11} CFU^{-1}
D	1.63997×10^{-6} CFU^{-1}
E	2.34733×10^{-8} CFU^{-1}

The plot for this function is shown as Fig. S1A along with the *in vivo* data.

For day 14 NTHi(WT) + Mcat(WT), the parameters are

Z	9.0914
A	7.85584×10^{-8} CFU^{-1}
B	4.35856×10^{-6} CFU^{-1}
C	-1.39511×10^{-10} CFU^{-1}
D	-4.51814×10^{-7} CFU^{-1}
E	2.47411×10^{-9} CFU^{-1}

The plot for this function is shown as Fig. S1B along with the *in vivo* data. This model applies to the $\{N_1, N_2\}$ domain from {0, 0} CFU to $\{530, 7\} \times 10^6$ CFU.

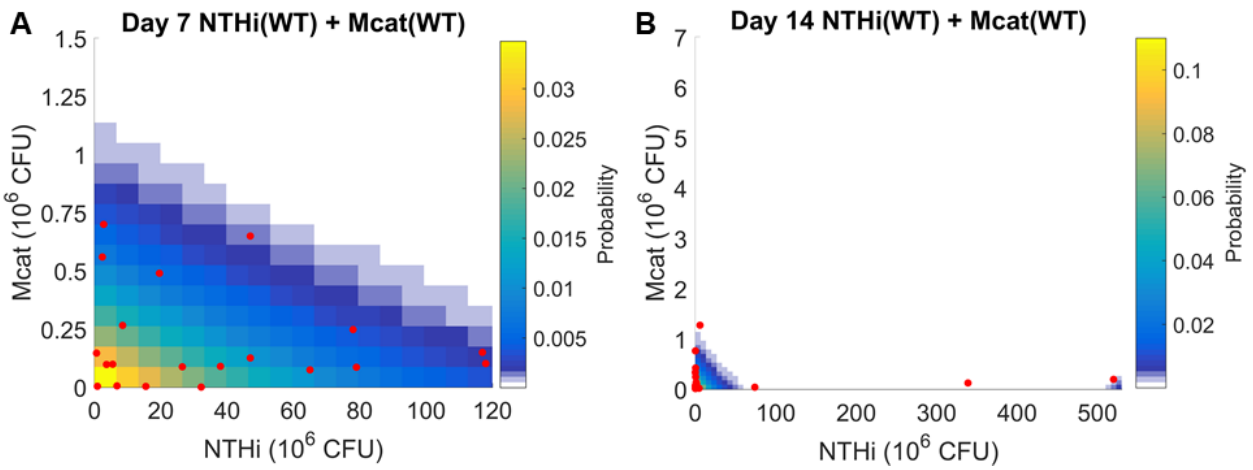

Figure S1. Host-host variations of NTHi (wt) and Mcat (wt) populations in co-infection experiments. (A) Shows populations of NTHi and Mcat in the counts (red points) obtained from the bullae of two ears of each animal for 10 chinchillas at day 7 post inoculation. The heat map shows the probability distribution function $\hat{p}(N_1, N_2)$ estimated by our MaxEnt based method. The axes have been zoomed into the region containing all *in vivo* data. **(B)** Measurements for day 14 post inoculation shown using the same visualization scheme as in (A). The heat map shows the MaxEnt estimation for $\hat{p}(N_1, N_2)$ corresponding to the data at day 14.

3. Derivation of Stability Analysis

Starting with Eq. (3), we have the following two equations describing the abundance of both species at steady state:

$$0 = N_1(1 - \alpha_{11}N_1 - \alpha_{12}N_2)$$
$$0 = N_2(1 - \alpha_{21}N_1 - \alpha_{22}N_2)$$

The stability matrix, *S*, which describes the derivative of each species with respect to all others near the steady state can be constructed. In general, for *n* total species, an element of *S* is given by

$$s_{ik} = \frac{\partial}{\partial N_k}\left(\frac{dN_i}{dt}\right)\Bigg|_{SteadyState}$$

$$s_{ik} = \frac{\partial}{\partial N_k}\left(N_i\left(1 - \sum_{j=1}^{n}\alpha_{ij}N_j\right)\right)$$

$$s_{ik} = \frac{\partial}{\partial N_k}(N_i)\left(1 - \sum_{j=1}^{n}\alpha_{ij}N_j\right) + (N_i)\frac{\partial}{\partial N_k}\left(1 - \sum_{j=1}^{n}\alpha_{ij}N_j\right)$$

The first term is *0*, because $\left(1 - \sum_{j=1}^{n}\alpha_{ij}N_j\right) = 0$ at Steady State.

$$s_{ik} = N_i\left(0 - \frac{\partial}{\partial N_k}\left(\sum_{j=1}^{n}\alpha_{ij}N_j\right)\right)$$

The second term is *0* for all *j*, except when *j=k*:

$$s_{ik} = N_i\left(-\frac{\partial}{\partial N_k}(\alpha_{ik}N_k)\right)$$

$$s_{ik} = -N_i\alpha_{ik}$$

For our two species system, the stability matrix can be written as

$$S = \begin{bmatrix} -N_1\alpha_{11} & -N_1\alpha_{12} \\ -N_2\alpha_{21} & -N_2\alpha_{22} \end{bmatrix}$$

We then calculate the Eigen values of *S*. The steady state is considered stable if and only if the Real parts of every Eigen value is less than or equal to 0.

4. Two parameter marginal distributions of $Q(\alpha_{11}, \alpha_{12}, \alpha_{21}, \alpha_{22})$

From the $P(N_1,N_2)$ fit of the reference data, we use a MaxEnt method to calculate $Q(\alpha_{11}, \alpha_{12}, \alpha_{21}, \alpha_{22})$. These four parameters correspond to the LV parameters (Fig. 1). α_{11} (>0) and α_{22} (>0) represent intra- species interactions for NTHi and Mcat, respectively. α_{12} and α_{21} represent the effect of Mcat on the growth of NTHi, and, NTHi on the growth of Mcat, respectively. α_{12} and α_{21} can be positive (competitive interaction), zero (neutral interaction), or, negative (co-operative interaction) based on Eq. (3). We calculate the six pairwise correlations and plot the marginal distributions to visualize these correlations. Fig. S2 shows the $Q(\boldsymbol{\alpha})$ for day 7, and Fig. S3 shows the $Q(\boldsymbol{\alpha})$ for day 14. These correlations between the α parameters have contributions from three sources: (i) the interdependence between them via Eq. (3), (ii) shape of the distribution of $P(N_1,N_2)$ and (iii) the stability criterion (Supplemental Section 3) that ensures that N_1 (>0) and N_2 (>0) in Eq. (3) are stable fixed points of the kinetics in Eq. (1).

	Mean
NTHi	36×10^6 CFU
Mcat	2×10^5 CFU
α_{11}	0.95×10^{-6} CFU^{-1}
α_{12}	-406×10^{-6} CFU^{-1}
α_{21}	-11.1×10^{-6} CFU^{-1}
α_{22}	762×10^{-6} CFU^{-1}

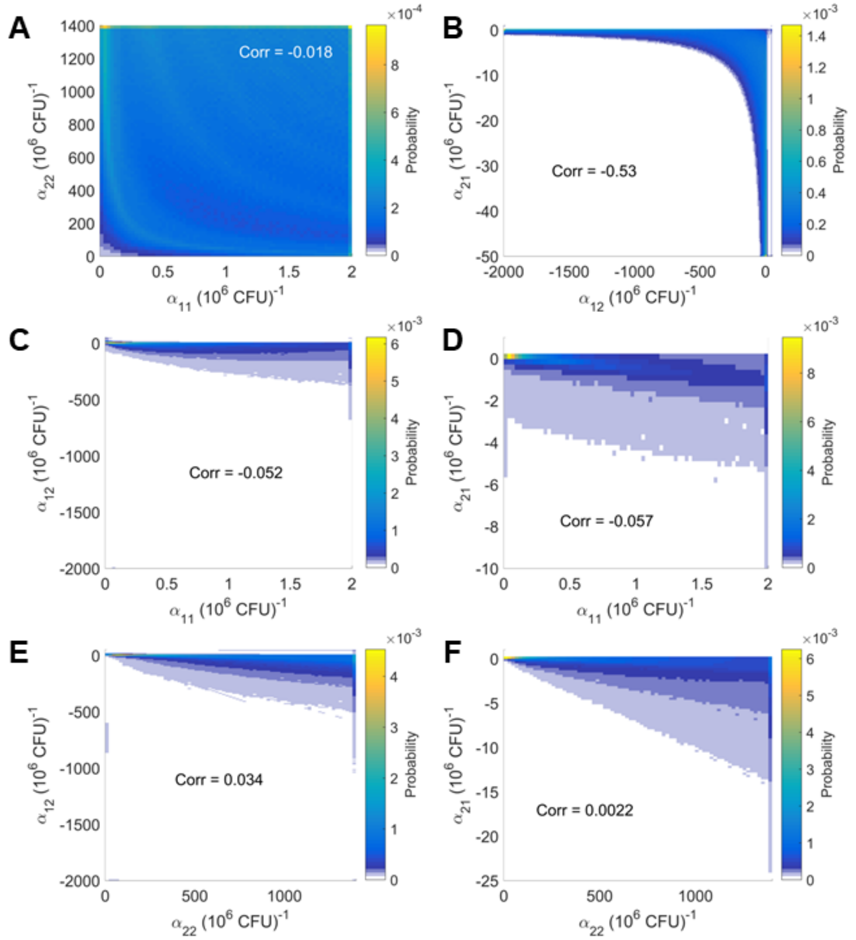

Figure S2. Day 7 Estimation of LV interactions in the co-infection kinects with wild type strains. The two dimensional joint distributions of the interactions, $Q(\alpha_{11}, \alpha_{12}, \alpha_{21}, \alpha_{22})$, are shown in six planes: **(A)** α_{11}-α_{22}, **(B)** α_{12}-α_{21}, **(C)** α_{11}-α_{12}, **(D)** α_{11}-α_{21}, **(E)** α_{22}-α_{12}, and, **(F)** α_{22}-α_{21} using heat-maps. The table shows the mean values of the populations of NTHi and Mcat and the interaction parameters. The Person correlations (Corr) between the interaction parameters are shown on the figures.

	Mean
NTHi	47×10^6 CFU
Mcat	2×10^5 CFU
α_{11}	0.97×10^{-6} CFU^{-1}
α_{12}	-325×10^{-6} CFU^{-1}
α_{21}	-16.5×10^{-6} CFU^{-1}
α_{22}	712×10^{-6} CFU^{-1}

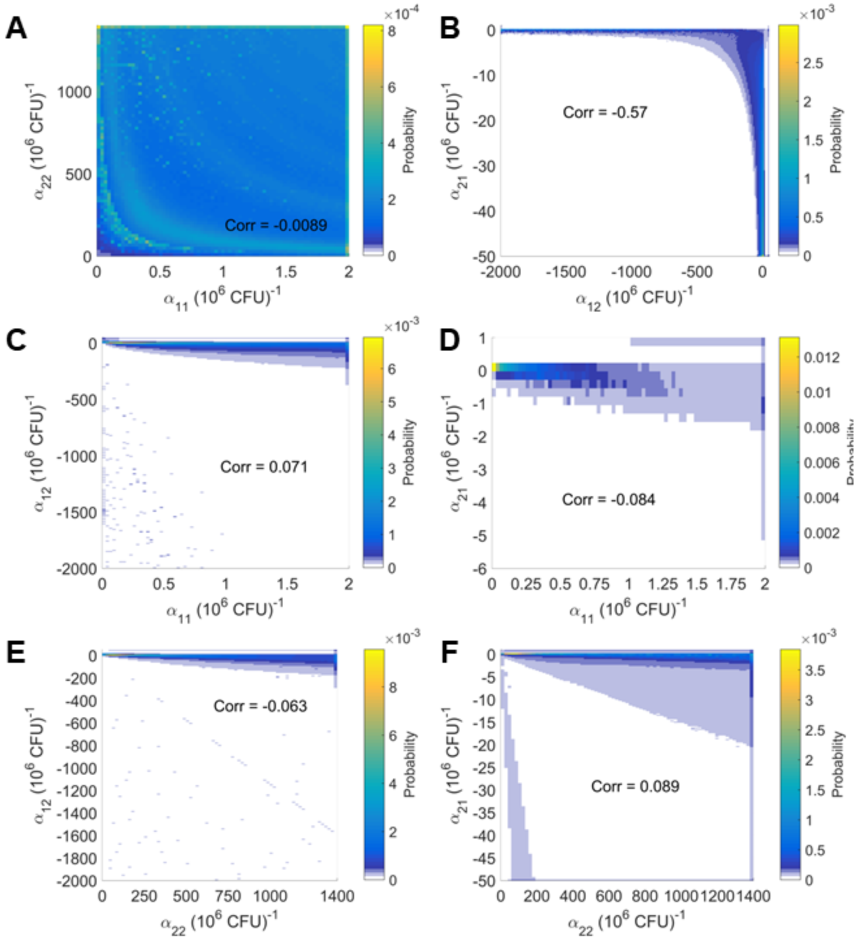

Figure S3. Day 14 Estimation of LV interactions in the co-infection kinects with wild type strains. The two dimensional joint distributions of the interactions, Q(α_{11}, α_{12}, α_{21}, α_{22}), are shown in six planes: **(A)** α_{11}-α_{22}, **(B)** α_{12}-α_{21}, **(C)** α_{11}-α_{12}, **(D)** α_{11}-α_{21}, **(E)** α_{22}-α_{12}, and, **(F)** α_{22}-α_{21} using heat-maps. The table shows the mean values of the populations of NTHi and Mcat and the interaction parameters. The Person correlations (Corr) between the interaction parameters are shown on the figures. This analysis is done using the same domains in NTHi-Mcat and **α**-space as the day 7 data.

5. Comparison between the Condorcet winner model and measurement at day 14 post inoculation with mutant strains

In the main text we present the results for bacterial kinetics as observed 7 days post co-infection. These results are presented with a model (called the Condorcet winner) which best describes the changes in the mutation strain data compared to wild type. We were also able to analyze data from 14 days post inoculation (Fig. S4).

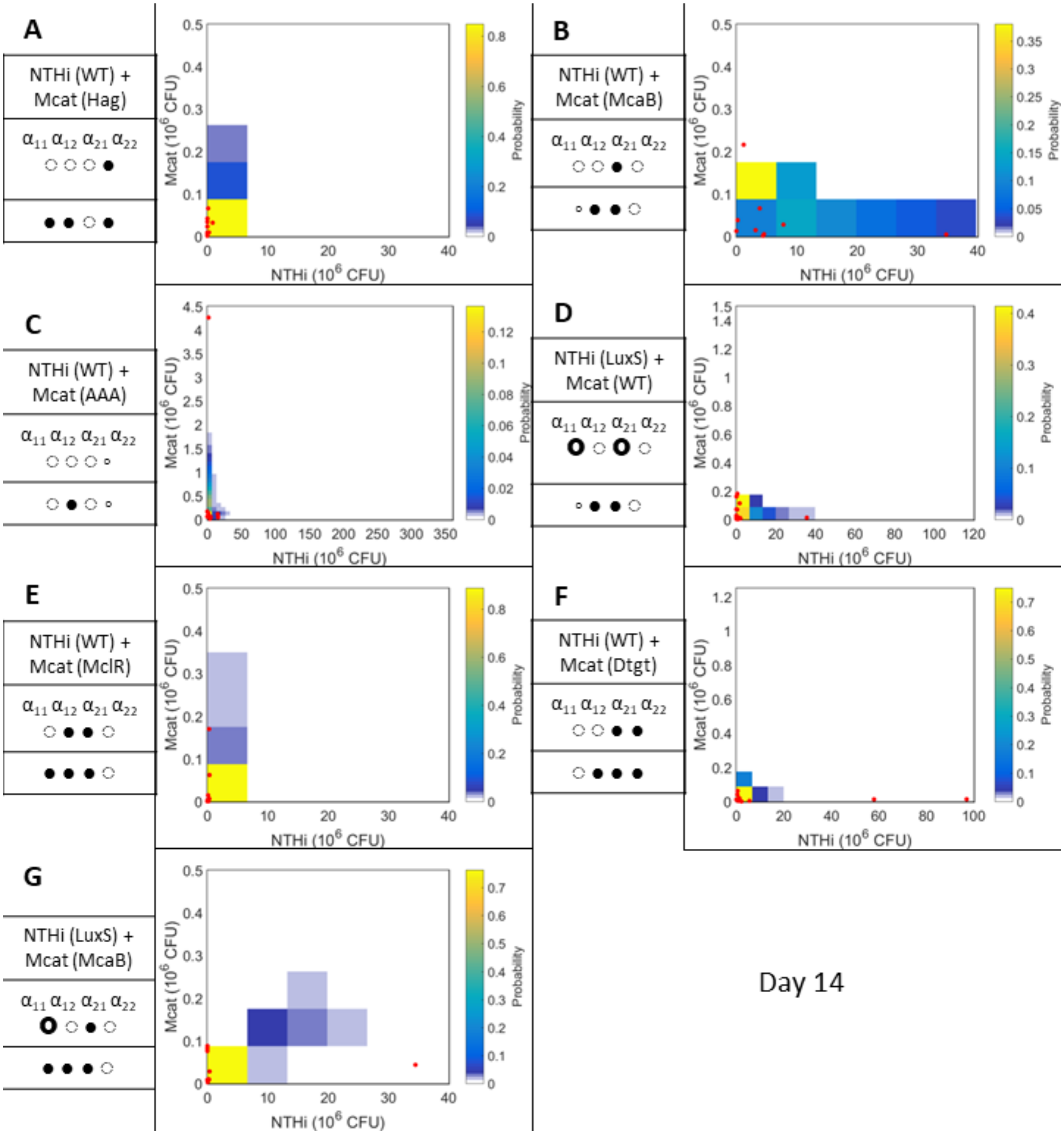

Figure S4. Day 14 post inoculation: data and predicted models. The data are displayed using the same visualization scheme as Fig. 4 in the main text. The following combinations are shown:

(A) NTHi(WT)-Mcat(*hag*). **(B)** NTHi(WT)-Mcat(*mcaB*). **(C)** NTHi(WT)-Mcat(*aaa*). **(D)** NTHi(*luxS*)-Mcat(*aaa*). **(E)** NTHi(WT)-Mcat(*mclR*). **(F)** NTHi(WT)-Mcat(*dtgt*). **(G)** NTHi(*luxS*)-Mcat(*mcaB*). All the mutant strains studied show unanticipated changes in the interactions *in vivo* that *strongly* regulate bacterial kinetics. The specific regulations are indicated by the Condorcet winning model. Fig. 4 shows at day 7, some strains' unanticipated changes are *weak* regulators of kinetics. This result indicates that the host immune response plays a more significant role in bacterial kinetics by day 14 than day 7.

6. Doubling the range of the α-domain does not change the results

As stated in the main text, we chose a fixed domain in α-space; that is, $\alpha_{11} \in [0.027, 2]$, $\alpha_{12} \in [-2000, 50]$, $\alpha_{21} \in [-50, 1]$ and $\alpha_{22} \in [18.9189, 1400]$. To verify that this chosen domain is sufficiently large and the results are independent of this choice, we repeated our analysis with a domain roughly twice as large (Fig. S5). That is, $\alpha_{11} \in [0.0541, 4]$, $\alpha_{12} \in [-4000, 100]$, $\alpha_{21} \in [-100, 5]$ and $\alpha_{22} \in [37.8378, 2800]$. The lattice size was proportionally increased with the domain.

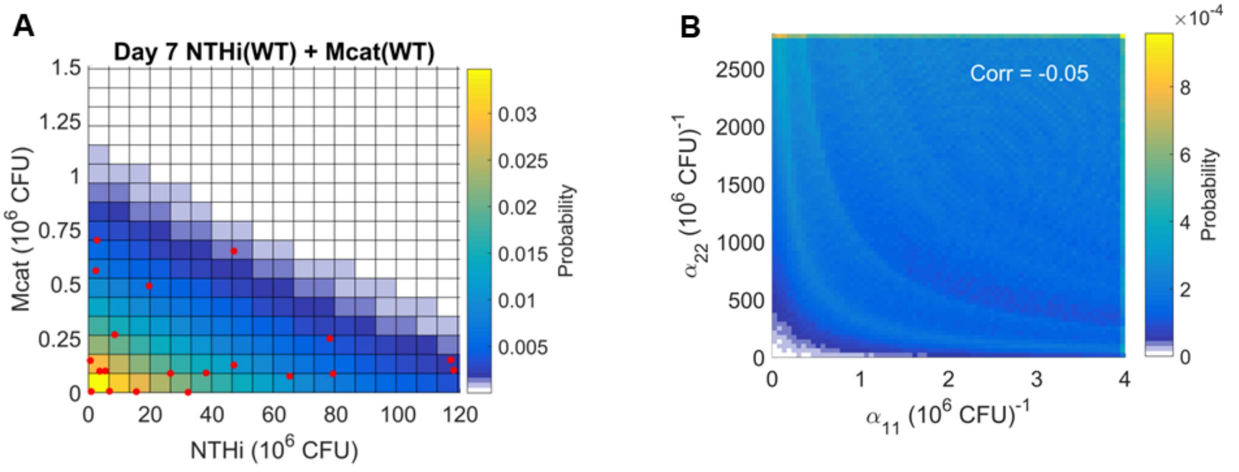

	Mean
NTHi	36×10^6 CFU
Mcat	2×10^5 CFU
α_{11}	1.96×10^{-6} CFU^{-1}
α_{12}	-809×10^{-6} CFU^{-1}
α_{21}	-22.3×10^{-6} CFU^{-1}
α_{22}	1597×10^{-6} CFU^{-1}

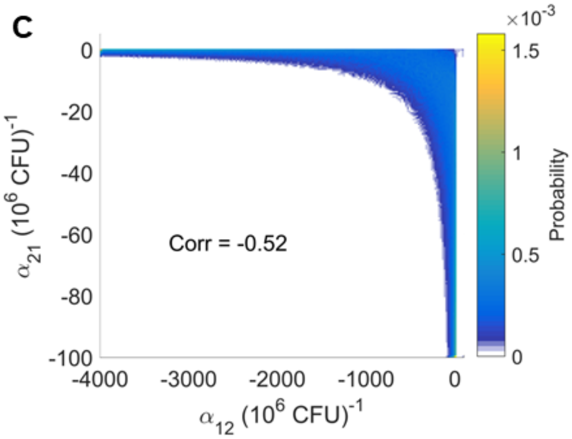

Figure S5. Doubled α-domain. (A) Shows the NTHi and Mcat populations at day 7 post inoculation along with the MaxEnt estimated $P(N_1,N_2)$. This panel is a reproduction of Fig. S1A. **(B)** and **(C)** show $Q(\alpha_{11}, \alpha_{22})$ and $Q(\alpha_{12}, \alpha_{21})$ respectively. These panels are analogous to Figs. S2A – S2B. Note that the correlations are similar, and because the domains were doubled in size, the average α-values are also doubled. Using this larger α-domain, we recalculated the predictions for the day 7 NTHi(WT) + Mcat(Hag) and day 7 NTHi(WT) + Mcat(McaB) co-infection experiment. The resulting predictions (data not shown) were equivalent to the ones shown in Figs. 4A – 4B.

7. *In silico* Reference Data

We generated three sets of *in silico* reference data; each estimated a specific level of the host's immune response. Below we show these three datasets (N_1, N_2) pairs, the $\hat{p}(N_1, N_2)$ fits and the corresponding $\hat{q}(\alpha_{11}, \alpha_{12}, \alpha_{21}, \alpha_{22})$.

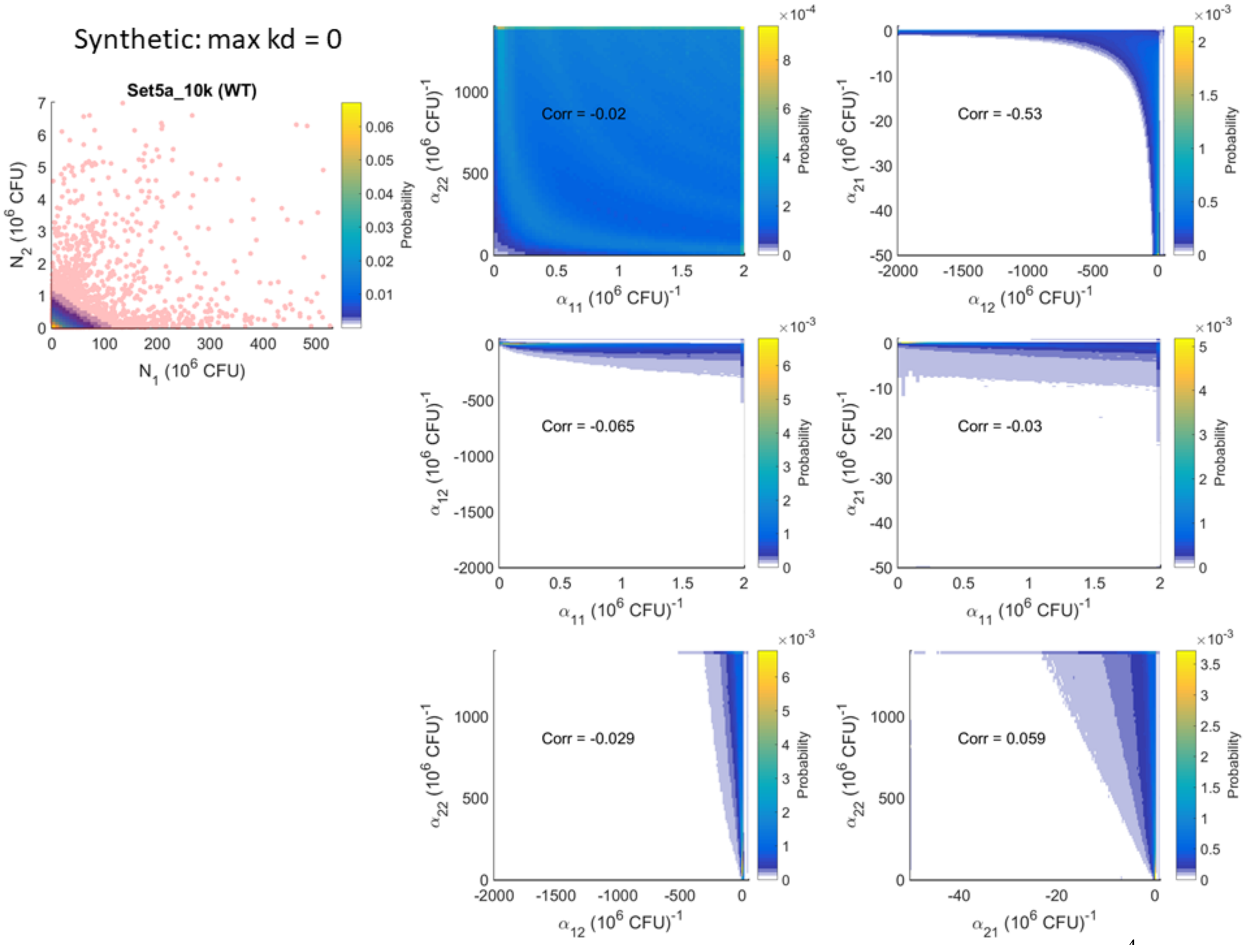

Figure S6. No Immune Term. In the absence of an immune response, we generated 10^4 pairs of (N_1, N_2) shown here as the red dots. The fit, $\hat{p}(N_1, N_2)$, is shown underlying the scattered points quantified using the color bar. For all six pair-wise combinations of the 4 parameters (α_{11}, α_{12}, α_{21}, α_{22}), we show the marginal distributions as in Fig. S2.

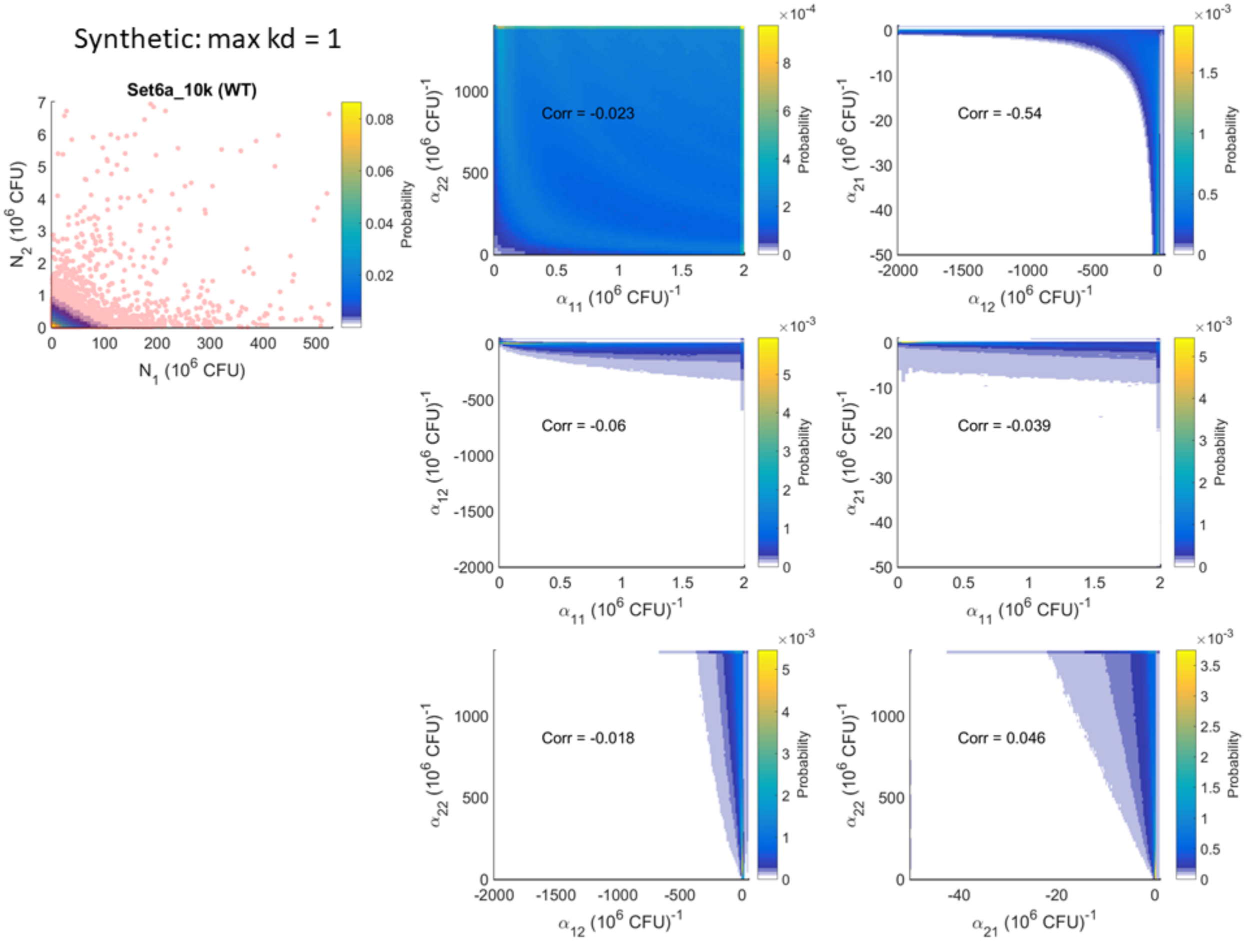

Figure S7. Weak Immune Term. To model a weak immune response, as described in the main text, we allow only N_2 to solicit an immune response, and only N_1 to be susceptible to inhibition by this response. This inhibition is weak (dictated by k_d). The panels are similar to Fig. S6.

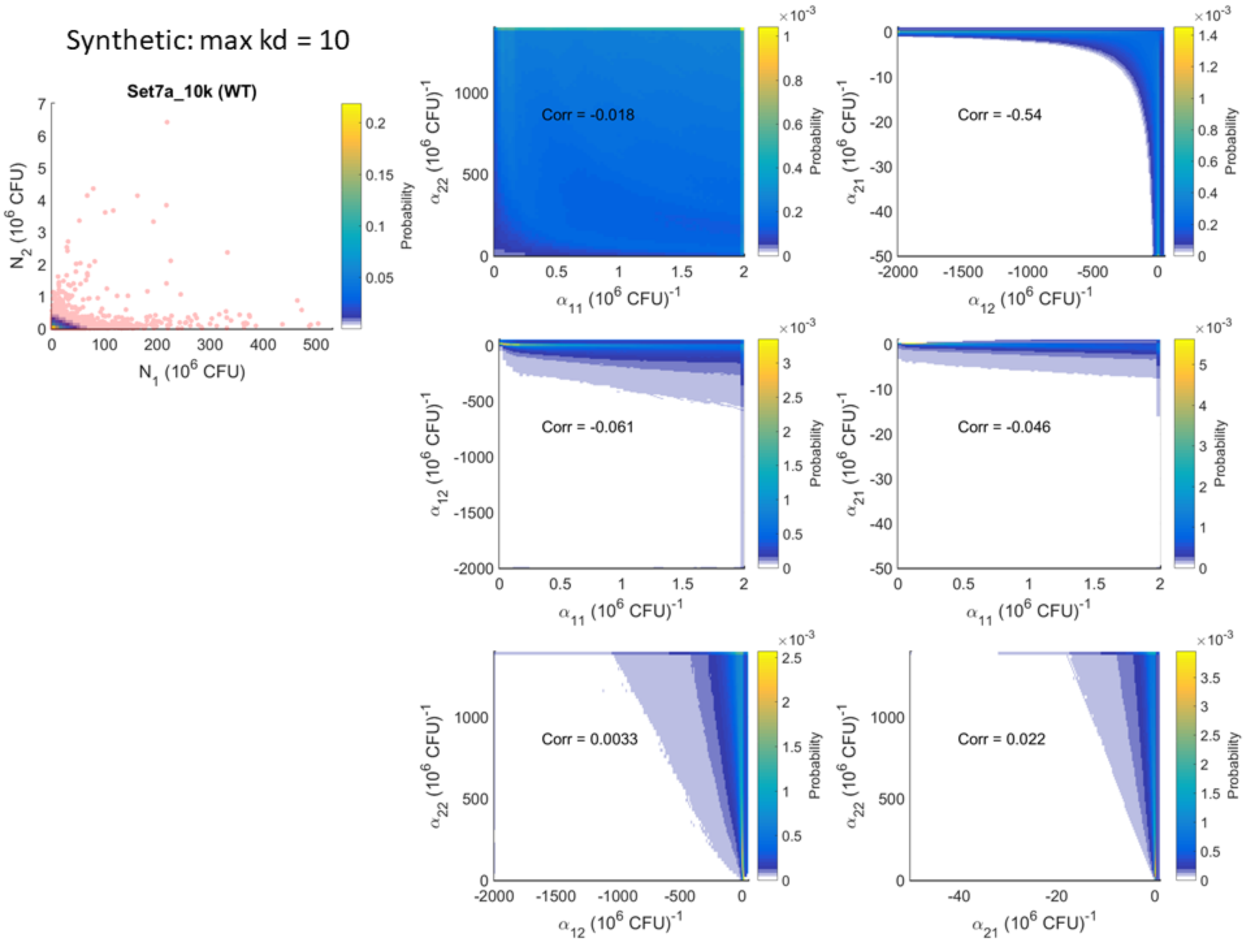

Figure S8. Strong Immune Term. Only N_2 solicits an immune response, and only N_1 is susceptible to inhibition by the immune response. This inhibition is strong (dictated by k_d). The panels are similar to Fig. S6.

8. Summary of all *in silico* mutant strain results

We generated 36 datasets *in silico* based on three levels of the immune term, three levels of the severity of the mutation and four mutations (one for each LV parameter). We applied our framework to each of these datasets and determined if the unanticipated changes in the interactions were *weak* or *strong* regulators of the bacterial kinetics. If *strong*, then we went further and identified the specific nature of the unanticipated regulation. Figs. S9 – S12 show these results. The winning models are denoted by the changes in $\{\alpha_{11}, \alpha_{12}, \alpha_{21}, \alpha_{22}\}$ for the wild-type+wild-type co-infection. O indicates no change, X indicates an increase, and, o indicates a decrease.

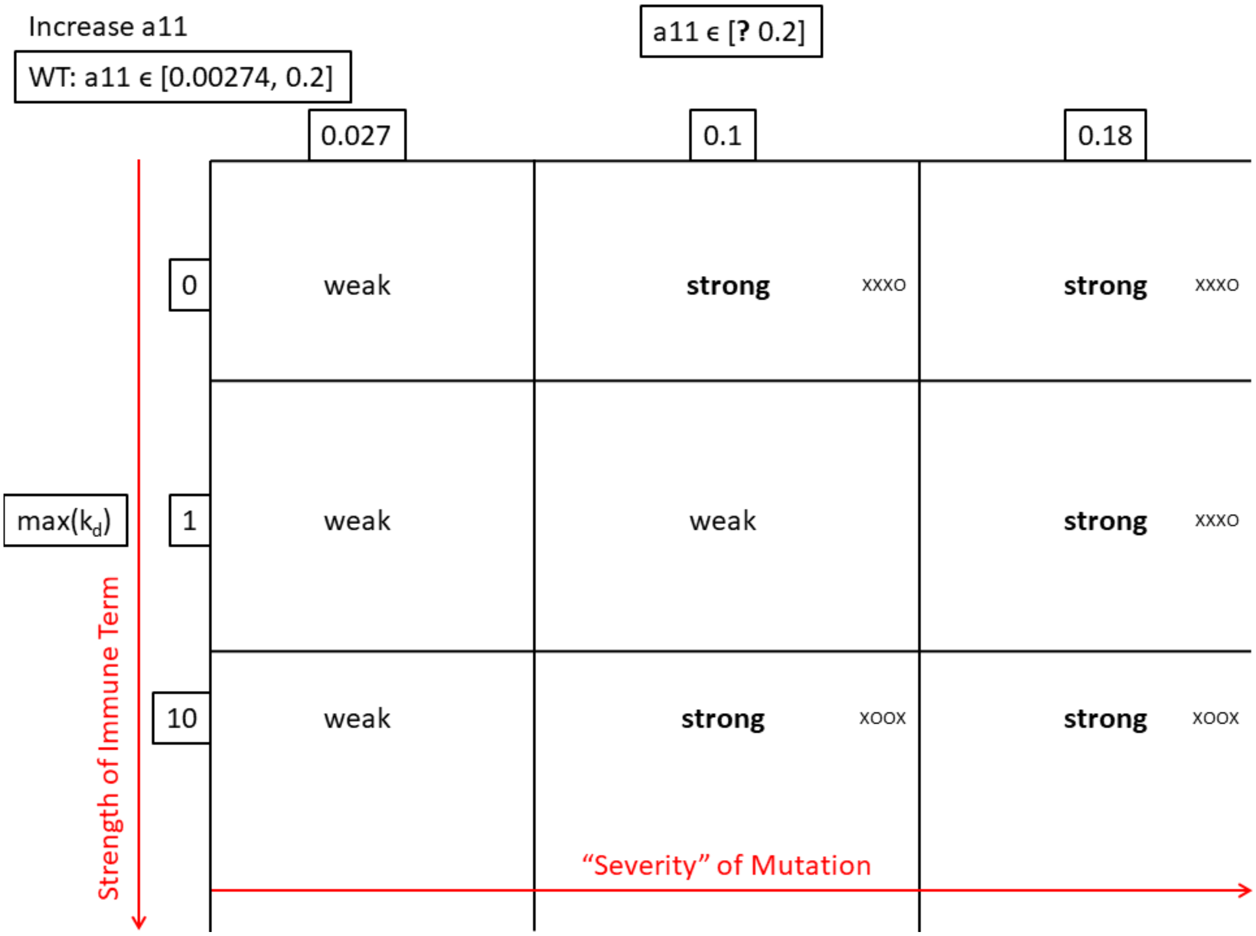

Figure S9. Mutation of α_{11}. We varied the strength of the immune term (increasing by row from top to bottom) and the severity of the mutation (increasing by column from left to right). The immune term is dictated by the k_d parameter (see Eq. (14)); the minimum is 0 and the maximum is indicated at each row. The severity of the mutation is dictated by the range of α_{11}; the wild type's range is shown on the top left. The values above the columns indicate the new minimum for each case.

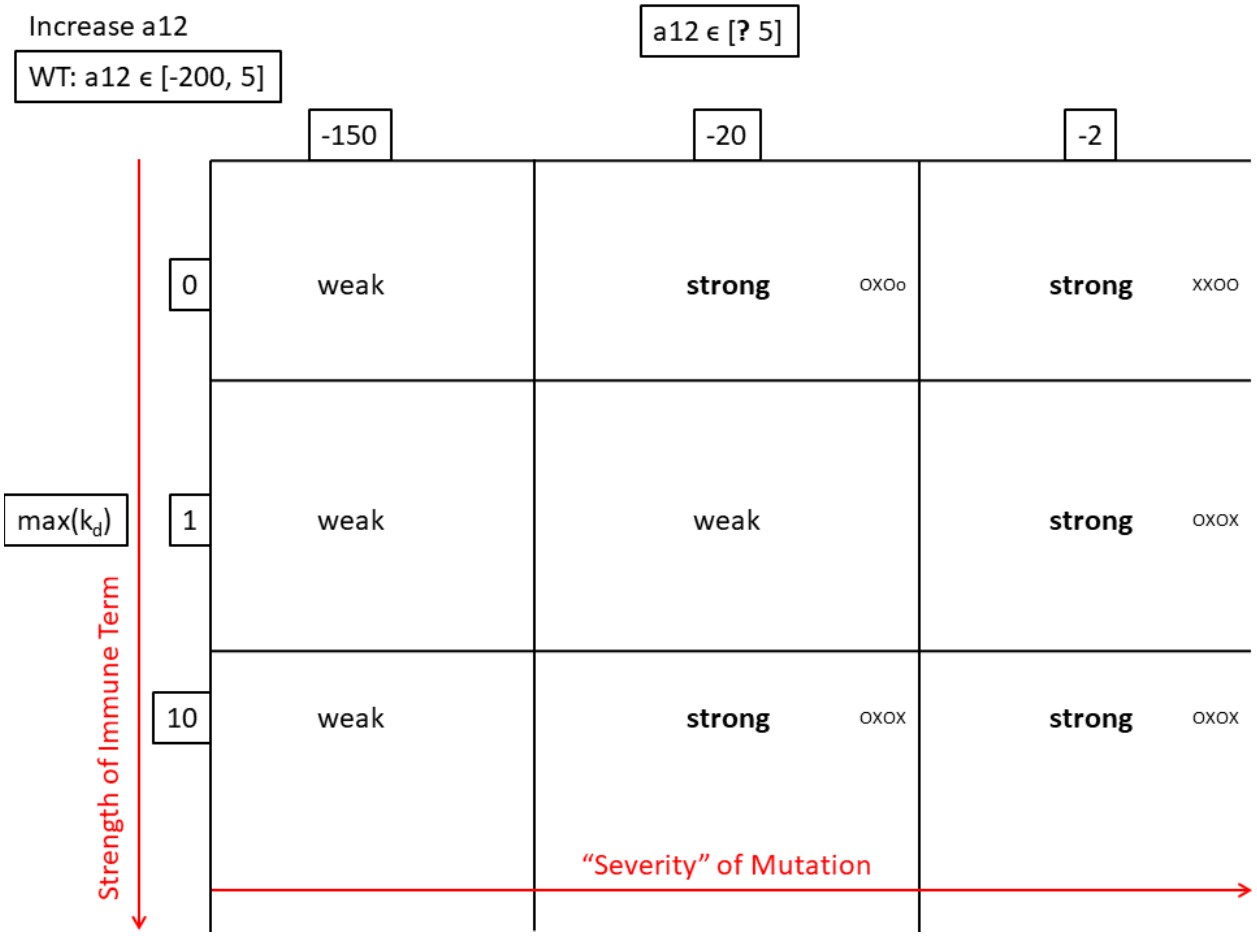

Figure S10. Mutation of α_{12}. The figure is arranged similar to Fig. S9. The severity of the mutation is dictated by the range of α_{12}; the wild type's range is shown on the top left.

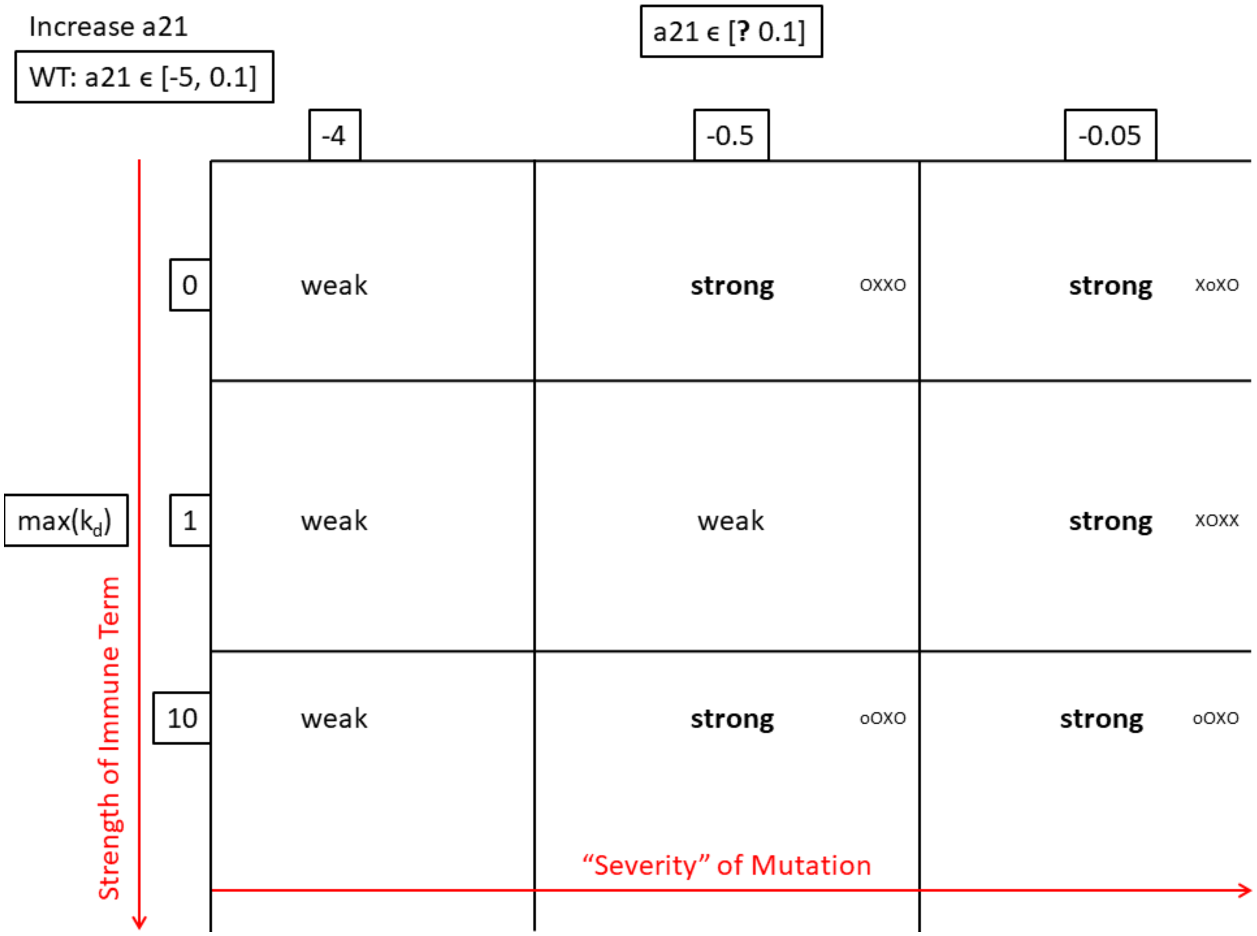

Figure S11. Mutation of α_{21}. The figure is arranged similar to Fig. S9. The severity of the mutation is dictated by the range of α_{21}; the wild type's range is shown on the top left.

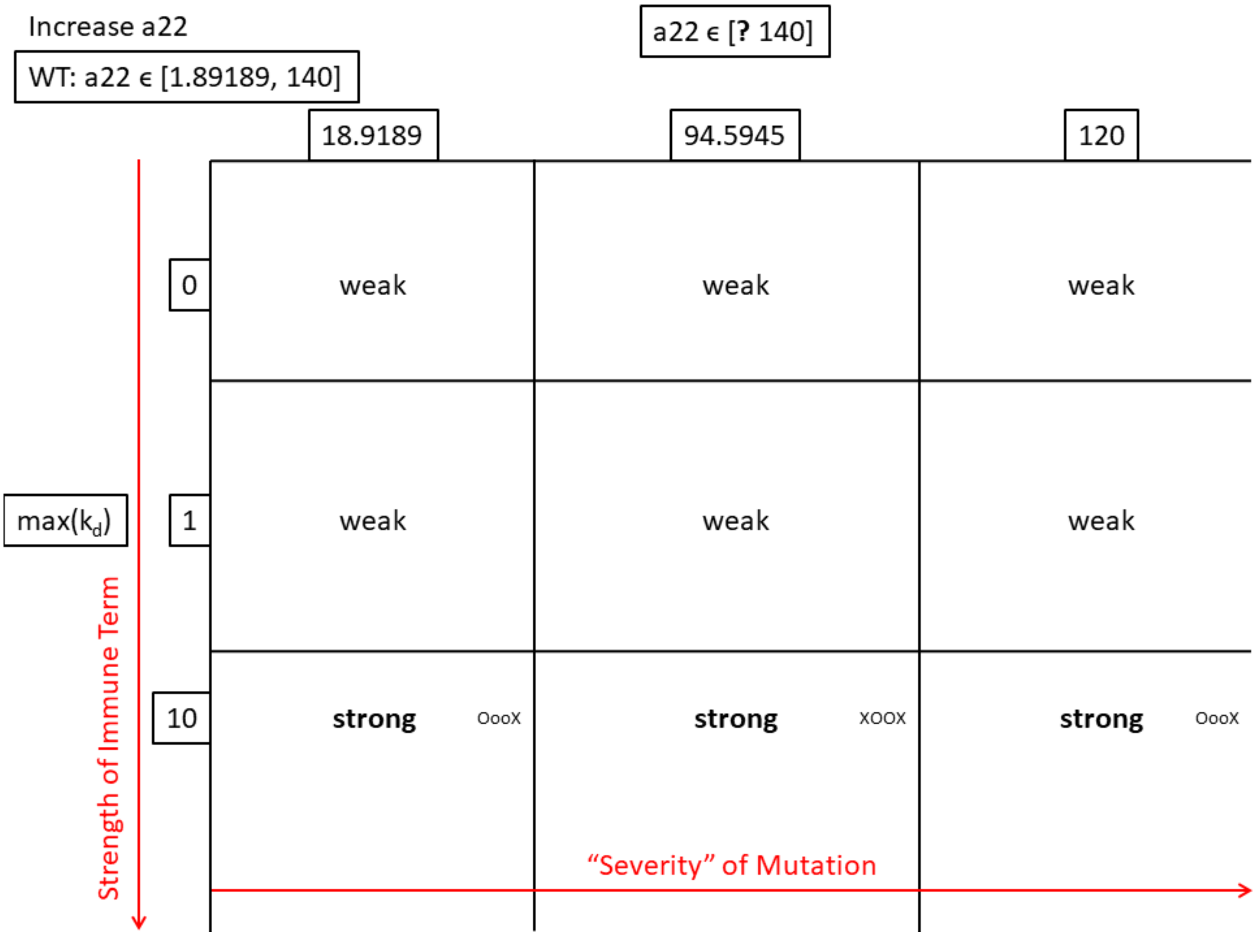

Figure S12. Mutation of α_{22}. The figure is arranged similar to Fig. S9. The severity of the mutation is dictated by the range of α_{22}; the wild type's range is shown on the top left.
